# Tracking humanitarian funding for reproductive health: a systematic analysis of health and protection proposals from 2002-2013

**DOI:** 10.1186/1752-1505-9-S1-S2

**Published:** 2015-02-02

**Authors:** Mihoko Tanabe, Kristen Schaus, Sonia Rastogi, Sandra K Krause, Preeti Patel

**Affiliations:** 1 Women’s Refugee Commission, 122 East 42 Street, New York, New York 10168, USA; 2 King’s College London, Department of War Studies, King's College London Strand, London WC2R 2LS, UK

**Keywords:** Reproductive health, family planning, gender-based violence, Financial Tracking Service, flash appeals, consolidated appeals process, Minimum Initial Service Package, conflict

## Abstract

**Background:**

The Inter-agency Working Group on Reproductive Health in Crises conducted a ten-year global evaluation of reproductive health in humanitarian settings. This paper examines proposals for reproductive health activities under humanitarian health and protection funding mechanisms for 2002-2013, and the level at which these reproductive health proposals were funded.

**Methods:**

The study used English and French health and protection proposal data for 2002-2013, extracted from the Financial Tracking Service (FTS) database managed by the United Nations Office for the Coordination of Humanitarian Affairs. Every project was reviewed for relevance against pre-determined reproductive health definitions for 2002-2008. An in-depth analysis was additionally conducted for 2009-2013 through systematically reviewing proposals via a key word search and subsequently classifying them under designated reproductive health categories. Among the relevant reproductive health proposals, counts and proportions were calculated in Excel based on their reproductive health components, primarily by year. Contributions, requests, and unfunded requests were calculated based on the data provided by FTS.

**Results:**

Among the 11,347 health and protection proposals issued from 345 emergencies between 2002 and 2013, 3,912 were relevant to reproductive health (34.5%). The number of proposals containing reproductive health activities increased by an average of 21.9% per year, while the proportion of health and protection sector appeals containing reproductive health activities increased by an average of 10.1% per year. The total funding request over the 12 years amounted to $4.720 billion USD, of which $2.031 billion USD was received. Among reproductive health components for 2009-2013 proposals, maternal newborn health comprised the largest proportion (56.4%), followed by reproductive health-related gender-based violence (45.9%), HIV/sexually transmitted infections (37.5%), general reproductive health (26.2%), and lastly, family planning (14.9%).

**Conclusion:**

Findings show that more agencies are responding to humanitarian appeals by proposing to implement reproductive health programs and receiving increased aid over the twelve year period. While such developments are welcome, project descriptions show comparatively limited attention and programming for family planning and abortion care in particular.

## Background

Access to reproductive health services is a human right [1]. Yet, lack of access to reproductive health information and services continues to cause excess morbidity and mortality for displaced women and girls in humanitarian settings [2]. The causes of poor reproductive health for conflict-affected populations are complex and multifold, including insufficient attention that certain components of reproductive health continue to receive despite concerted advocacy [3]. A 2009 study published by Patel et al. found that among total official development assistance (ODA) disbursed to 18 conflict-affected countries in 2003-2006, only 2.4% was allocated to reproductive health-related activities and services, of which a mere 1.7% was dedicated towards family planning activities [4]. Another study by Hsu et al. in 2013 found a slight increase in aid for reproductive health for 2009-2010 and a steady contribution overall; yet, due to discrepancies in funding across reproductive health activities, the authors note the need to examine resource allocations across activities and to encourage donors to target aid to those most in need [5].

One complementary way of tracking reproductive health assistance in humanitarian settings is through reviewing project and funding data that are reported to the United Nations Office for the Coordination of Humanitarian Affairs’ (UN OCHA) Financial Tracking Service (FTS); a database that contains up-to-date project and donor information. The FTS is a global, real-time database that records all reported international humanitarian aid from UN agencies, non-governmental organizations (NGOs), the Red Cross/Red Crescent Movement, bilateral aid, in-kind assistance, and private donations to crises where appeals have been launched. Appeals are primarily launched when needs exceed the ability of the government and any one agency to respond adequately to a conflict, natural disaster, or prolonged crisis [6]. The FTS primarily focuses on flash and consolidated appeals [7]: the Flash Appeal is a tool for structuring a coordinated humanitarian response to address urgent life-saving needs in the first three to six months of an emergency. If the emergency continues beyond six months, the Flash Appeal may be developed into a Consolidated Appeal (CAP) of up to 12 months. The CAP includes the Common Humanitarian Action Plan (CHAP), which is a strategic plan for humanitarian response in a specific country or region [8]. During appeal development, cluster/sector coordinators are responsible for gathering project proposals. They lead the peer review process of vetting proposals; issued and listed appeals therefore only include approved proposals, although agencies can modify projects as needs evolve [7]. FTS data are provided by donors and recipient organizations and include 1-2 page project summaries that are publicly available [9].

In 2012, the Women’s Refugee Commission (WRC) used FTS data to examine the extent to which appealed health projects included reproductive health services for adolescents aged 10-19 years. Findings showed that less than 3.5% of all health proposals in any given year included a component of adolescent reproductive health, and among them, only 32% received any related funding [10]. Such methods of analysis are invaluable to tracking progress and measuring the impact of reproductive health-related advocacy.

As part of the Inter-agency Working Group (IAWG) on Reproductive Health in Crises’ ten-year global evaluation of reproductive health in humanitarian settings, the WRC embarked on an analysis to examine for 2002-2013: 1) the extent to which humanitarian and development agencies, as well as local actors, have proposed to implement various reproductive health activities in humanitarian health and protection appeals; and 2) the level at which these reproductive health proposals were funded. This study complements a follow-up study undertaken by Patel et al. that examines longer-term trends in patterns of ODA for reproductive health activities in conflict-affected countries for the years 2002-2011 [11]. The FTS study was undertaken since ODA analysis of funding towards gender-based violence (GBV) programs is not possible due to a lack of a purpose code in the Creditor Reporting System (CRS) to which ODA is mandatorily reported by bilateral donors under pre-set aid categories. In addition, while the CRS provides conflict-affected countries as the unit of analysis, the FTS presents the ability to determine projects and funds that are directly availed to specific emergencies within countries, albeit reported by implementing agencies and donors in a voluntary manner [12]. This enables a closer examination of projects and funding that directly targets emergencies, overcoming assumptions that all ODA to a conflict-affected country in fact reaches the conflict-affected location. Despite several articles that employed CRS data to track funding flows to health and other relevant humanitarian topics [13,14], the authors found only one article in the literature that systematically analyzed FTS data for mental health and psychosocial support initiatives [15]. In the gray literature, CARE International has conducted a review of FTS appeals from 17 countries to examine donor spending on gender in emergencies as indicated by scores from the gender marker [16]. Our study therefore aimed to contribute to the literature on programming and funding for reproductive health in humanitarian settings, through conducting a comprehensive and systematic analysis of health and protection proposals for the years 2002-2013.

## Methods

### Data source

All data used in this study were extracted from OCHA’s FTS (http://fts.unocha.org/). More specifically, in August 2013, the WRC extracted health and protection project data from every conflict, natural disaster, or protracted crisis where a Flash, CAP, or other appeal was launched between 2002 and 2012 from FTS’ country-specific excel spreadsheets: “E. List of Appeal Projects (grouped by Cluster) with funding status of each” (Spreadsheet E). This was further supplemented by custom tables that could be created through following the FTS’s “Funding and Requirements by Project” tool where necessary [17]. Appeals from 2013 were extracted in March 2014. Appealed projects included those published in both English and French; the two languages for which appeals are available.

The data points from all emergencies over the 12 year period were compiled into a master Excel file where only appeals from Health and Protection were kept for analysis.^a^ In addition to Spreadsheet E, for 2009-2013, the study team downloaded all hyperlinked pdf “Project Descriptions” (project proposals) accessible from Spreadsheet E’s “Project Code” column. Since not all project descriptions were publicly available from years prior to 2009, the study retrieved OCHA’s accompanying comprehensive narrative appeals for 2005-2008 in lieu of the project proposals. For 2002-2003, only Spreadsheet E was extracted given the lack of availability of project descriptions and narratives. Spreadsheet E was thus the common data source for all 12 years.

### Analysis

Two WRC staff (KS and SR) analyzed the health and protection proposals; one of whom analyzed those from 2009 to 2010; the other the remaining ten years. They conducted in-depth analysis of health and protection proposals for 2009-2013 since all pdf proposals were available. The team assessed the content of each proposal by clicking the hyperlinked pdf “Project Descriptions” and conducting systematic key word searches within the activities and indicators sections of each proposal.^b^ Terms used included “repro,” “MISP,“ maternal,” “preg,” “family planning,” “condom,” “sex” (for sexual violence, etc.), “gender” (for gender-based violence, etc.), “STIs” (for sexually transmitted infections), “adolesc” (for adolescents/adolescence),“youth,” among others. Where seemingly relevant proposals were identified, the team read the proposals to ensure that key words were nuanced appropriately and related activities were not missed if other terms were used to address possible political sensitivities. If key words were only mentioned in the background or needs sections, the proposals were omitted from the tallies since they were considered less likely to actually implement relevant activities than those that contained key words as part of their activities or indicators.

For each relevant proposal, the analysis team categorized activities according to pre-determined definitions of reproductive health components. The definitions modeled distinctions made between the Minimum Initial Service Package^c^ (MISP) for reproductive health—the international minimum standard of care for reproductive health in emergencies—and more comprehensive reproductive health per the *Inter-agency Field Manual on Reproductive Health in Humanitarian Settings* (IAFM) [18]. Activities were further categorized into thematic components; namely: maternal newborn health; family planning; sexually transmitted infections (STIs), including HIV; gender-based violence (GBV); and general reproductive health. GBV findings were additionally categorized as reproductive health-related activities and non-reproductive health-related activities. Reproductive health-related GBV activities included clinical interventions, as well as those outlined in the IAFM to be within the scope of reproductive health. Non-reproductive health-related GBV activities included legal justice, protection, security, livelihoods, and gender, among other complementary interventions. A detailed categorization of reproductive health activities is listed in Table 1.

**Table 1:** Categorization of reproductive health activities per the IAFM [18]

Topic	MISP	Comprehensive reproductive health (excluding MISP activities)	Non-reproductive health
Maternal newborn health	• Emergency obstetric and newborn care services, including post-abortion and safe abortion care. • 24/7 referral system for obstetric and newborn emergencies. • Clean delivery packages to visibly pregnant women and birth attendants. • Informing communities about services.	• Antenatal care (ANC). • Post-natal care. • Breastfeeding promotion. • Training skilled attendants (midwives, nurses, doctors) in performing EmOC and newborn care.	• Nutrition outside of ANC.

Family planning	• Contraceptives to meet demand, such as condoms, pills, injectables, and intrauterine devices.	• Comprehensive family planning programming, including provision of long-term and permanent methods. • Community education. • Contraceptive supply chain management. • Staff training for family planning.	N/A

STIs/HIV	• Safe and rational blood transfusion practice. • Adherence to standard precautions. • Free condoms. • Syndromic treatment for STIs. • Antiretroviral (ARV) treatment for patients already taking ARVs. • Prevention of mother-to-child transmission.	• Comprehensive STI prevention and treatment. • STI surveillance systems. • Comprehensive HIV prevention, care, and treatment. • Staff training for HIV/AIDS.	N/A

GBV	• Sexual violence coordination within health sector/cluster mechanisms. • Physical protection and strategies for safe access to health facilities, including lighting and locks on latrines; prevention of sexual exploitation and abuse; codes of conduct; standard operating procedures. • Clinical care for survivors of sexual violence, including emergency contraception, post-exposure prophylaxis, etc. Forensic evidence is also included if applicable. • Other response services including psychosocial and mental health services. • Referrals to sexual violence services. • Informing communities about services.	• Prevention of domestic violence, forced early marriage, female genital cutting/mutilation. • Engaging men and boys, primarily to enhance access to RH for women and girls. • Staff training for clinical GBV.	• Multi-sectoral GBV coordination. • Legal justice • Protection • Child protection • Livelihoods • Security • Education • Empowerment • Gender • Trafficking • Unspecified “protection” activities • GBV Information Management System

General reproductive health	• RH coordination, including identifying an RH officer; holding coordination meetings; reporting back to the health cluster/sector. • Procurement of RH kits and supplies. • Planning for comprehensive RH, including collecting MISP and background data; identifying sites for future delivery of comprehensive RH; assessing staff capacity and planning trainings; and procuring RH supplies. • Disaster risk reduction. • Menstrual hygiene; dignity kits.	• General staff trainings for unspecified topics. • Routine RH procurement for unspecified topics. • Routine data collection beyond MISP indicators. • Cervical cancer screening and treatment. • Fistula repair. • Treatment of female genital mutilation/cutting complications. • Other gynecological services. • Unspecified RH activities.	N/A

**Figure 1:**
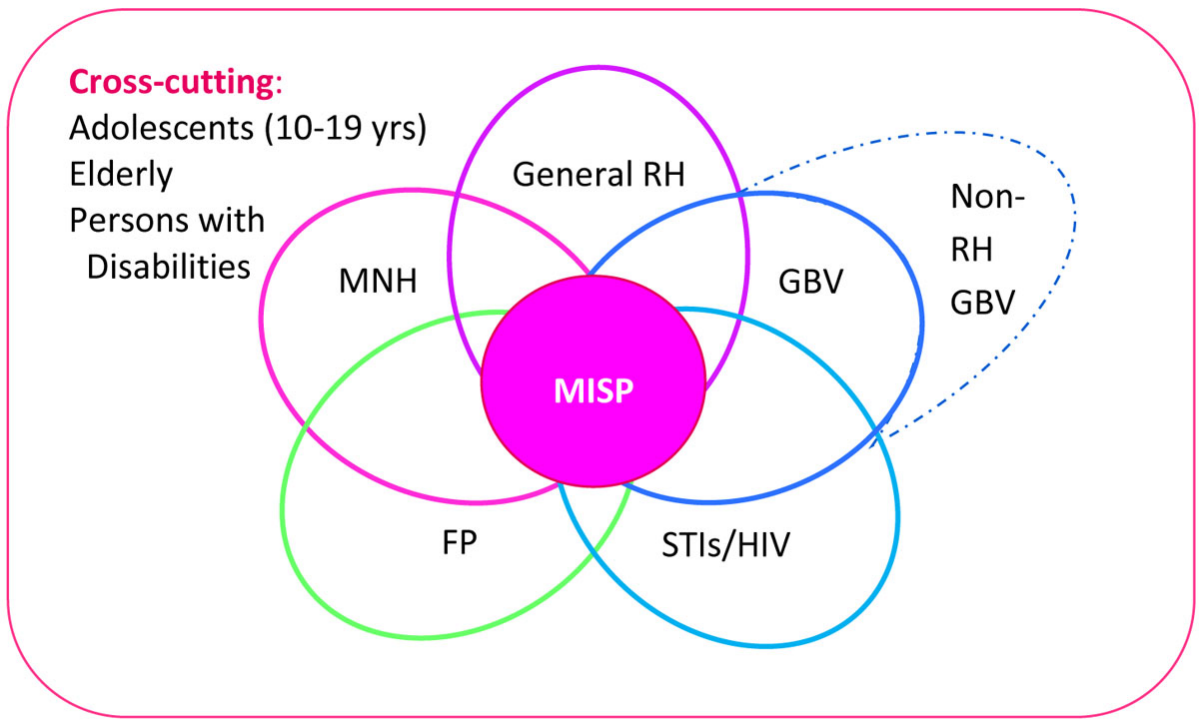
Relationshop between Reproductive Health Components.

As each proposal was 1-2 pages, some discretion was made on the part of the analysis team on how to categorize activities. In general, if the “MISP” was mentioned, it was assumed for categorization purposes that agencies implemented the standard in its entirety. While in reality, many reproductive health activities overlap in terms of their thematic categories—such as the prevention of mother-to-child transmission of HIV (PMTCT) as part of HIV and maternal newborn health services—to prevent inflated and duplicative counts, activities were categorized mutually exclusively into the five thematic reproductive health components per the MISP and comprehensive reproductive health (minus the MISP). A pictorial representation of the five reproductive health categories is noted in Figure 1.

For 2002-2008 appeals, given limited availability of publicly accessible information on FTS, a more cursory analysis was conducted to determine the projects’ relevance to reproductive health. Projects funded from 2005 to 2008 were deemed relevant if their titles or accompanying comprehensive narrative reports—generated by OCHA on behalf of the humanitarian system—mentioned reproductive health components or related activities. For 2002-2005, due to the additional lack of accessible comprehensive narratives, projects were coded as relevant if titles—which were the only specific column available on Spreadsheet E—mentioned reproductive health components or related activities. Taking into account the documented relative lack of attention to reproductive health in the earlier years as compared to later years [2], the study team included the earlier years despite some possibility of under-identification of relevant projects where only Spreadsheet E was available.

Among the relevant reproductive health proposals, counts and proportions were calculated in Excel based on their manual categorizations, primarily by year. Contributions, requests, and unfunded requests were calculated based on the data provided in Spreadsheet E. The total request per year was determined by summing spreadsheet E’s “Revised requirements USD” column. The total request funded was found by summing “Funding USD,” and the total unfunded request was calculated by summing “Unmet requirements USD”. For 2009-2013, funds from these columns were further divided by the number of reproductive health components that were encompassed in each proposal and then summed to calculate total request, total request funded, and total unfunded request per reproductive health component.

Among the projects analyzed, duplicate proposals in the context of revised appeals were included to take into account program evolution over time. Duplicate proposals arising from multiple agency requests for the same project were also included given the impossibility of de-linking projects without additional information and verification processes with appealing agencies. Withdrawn or blank proposals were additionally included for analyses; however, this had no impact on funding calculations since those columns from FTS were blank.

In order to complement Patel et al.’s ODA analysis, this study similarly grouped proposals according to the 18 conflict-affected countries and all other countries where appeals were launched. The 18 countries included Afghanistan, Angola, Burundi, Central African Republic, Chad, Colombia, Democratic Republic of Congo, Eritrea, Iraq, Liberia, Myanmar, Nepal, Sierra Leone, Somalia, Sri Lanka, Sudan, Timor-Leste, and Uganda. As Patel et’ al.’s study included South Sudan as part of the “Sudan” category, this study also added South Sudan to the list of 18 original countries from 2011 when distinctions between Sudan and South Sudan were made in the FTS. Where regional appeals were launched—West Africa in particular—as country-specific activities and funds could not be extracted, these appeals were excluded from the 18 country count.

## Results

Findings

### Overall findings for 2002-2013

In total, 11,347 health and protection proposals from 345 emergencies were issued between 2002 and 2013. The major humanitarian emergencies during this time included: crises in the Southern African region (2002-2003), Indian Ocean earthquake and tsunami (2005), earthquake in Haiti (2010), floods in Pakistan (2011), the Syria conflict (2012-2013), and food insecurity in Sub-Saharan Africa throughout the decade. Among the 11,347 proposals, the study identified 3,912 proposals that were relevant to reproductive health, which amounted to 34.5% of all health and protection proposals combined.

**Figure 2:**
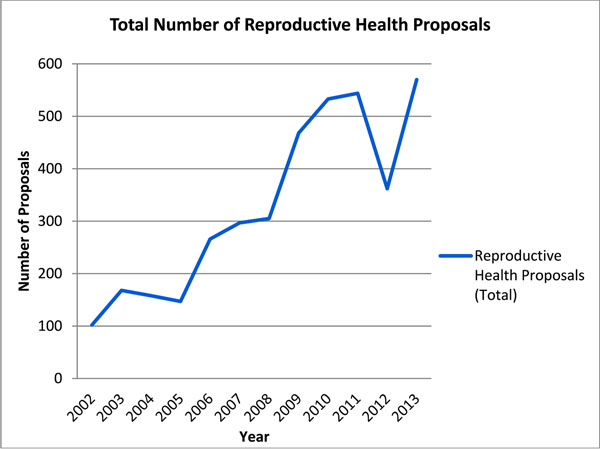


**Figure 3:**
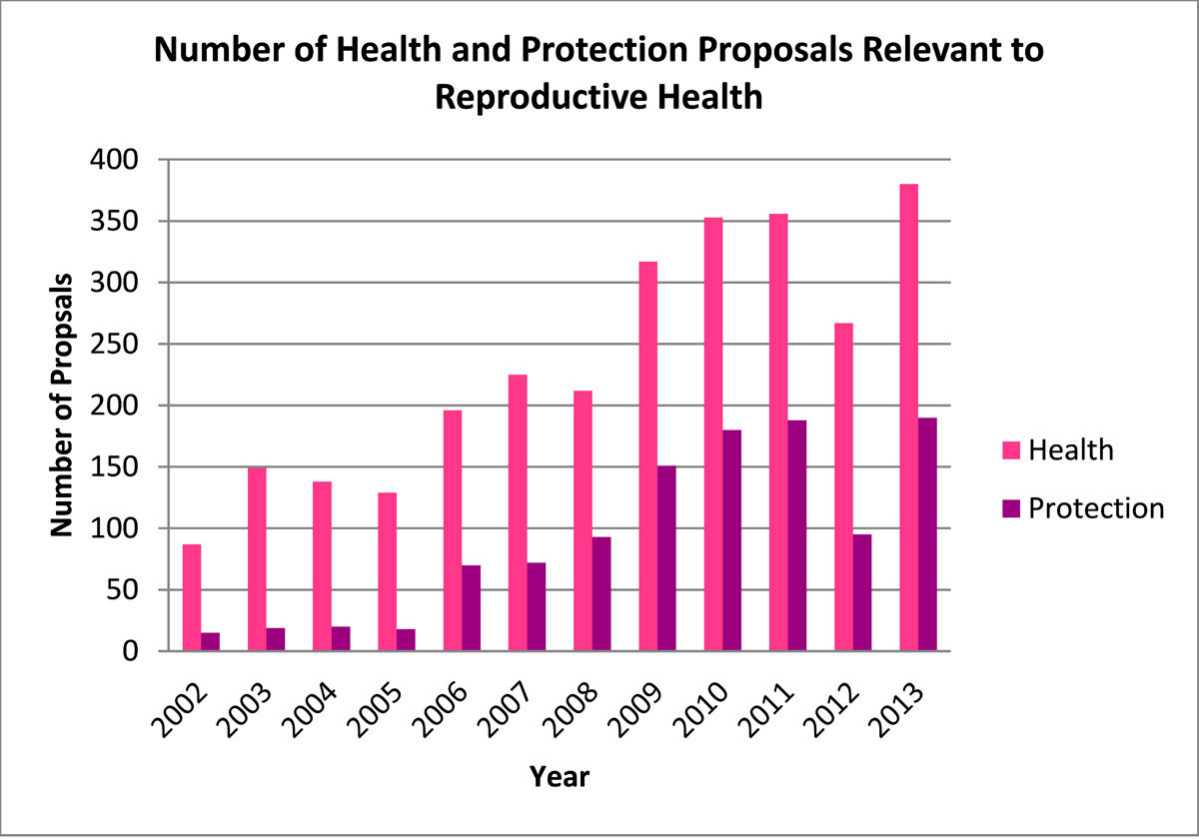


From 2002 to 2013, there was a 136.4% increase in the number of proposals requesting funding under Health and a 200.8% increase in the number of proposals requesting funding under Protection. This amounted to an average annual increase of 9.8% and 13.3%, respectively. By contrast, during the two time intervals, the number of health and protection proposals that addressed some component of reproductive health increased by 336.8% and 1,166.7%, respectively. This reflected a combined average annual increase of 21.9%, or 17.9% for Health and 43.6% for Protection over the 12 year period. In terms of proportions, reproductive health accounted for 34.5% of health and protection sector proposals, with an average annual increase of 10.1% per year. In terms of absolute counts, 2002 marked the lowest number of relevant reproductive health projects, with 87 identified for Health and 15 for Protection. On the other hand, while a drop was observed in 2012, the general trend showed progressive increases, with 2013 marking the highest number of relevant reproductive health proposals (570). In 2013, 380 health and 190 protection proposals were identified as relevant to reproductive health (53.4% of total health and protection proposals). See Figures 2 and 3 for details.

**Figure 4:**
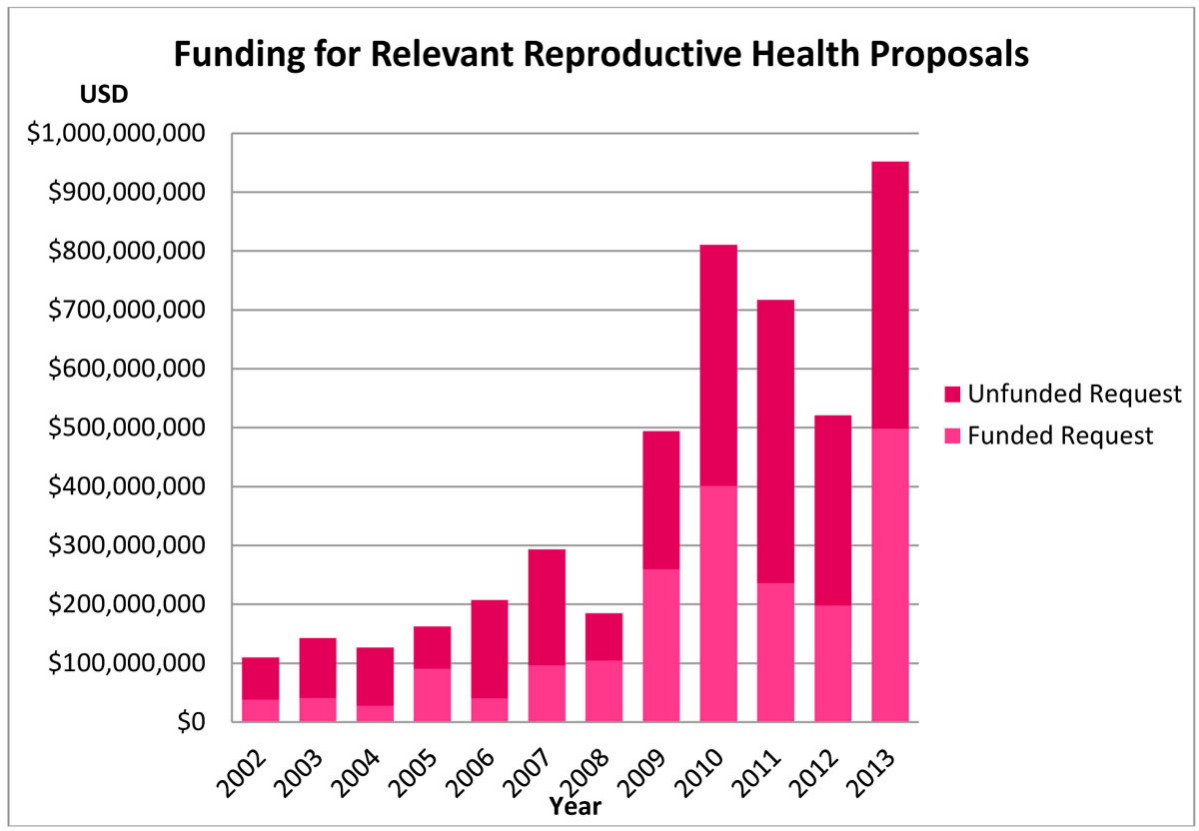


Funding requests from the relevant reproductive health projects over the 12 years amounted to $4.720 billion USD. From this, $2.031 billion USD was received, with an unfunded request of $2.689 billion USD (57.0%). Hence, 43.0% of the total request was funded, with an average of 39.6% of the request funded per year. Reproductive health projects were least funded in 2006 (19.5%), and most funded in 2008 (56.5%). The overall change in funding requests from 2002 to 2013 was 771.5%, with a 17.9% average annual increase in the proportion of requests received per year. Absolute amounts further showed that funding towards reproductive health projects increased from approximately $38.3 million in 2002 to $498.3 million in 2013, which was 35.0% and 52.3% of the funding request in 2002 and 2013, respectively. See Figure 4 for more information.

### In-depth analysis for 2009-2013

For the five year period from 2009 to 2013, a total of 5,636 proposals were filed for the two sectors, which comprised 3,358 health and 2,278 protection proposals. Among these, 2,477 were relevant to reproductive health (43.9%). From 2009 to 2013, a decrease in the number of issued health and protection proposals was observed by 15.5% and 34.4%, respectively. This translated to an average annual decrease of 2.8% and 9.3% for health and protection proposals, respectively. On the contrary, the share of relevant reproductive health proposals increased for both sectors, with overall and average annual increases of 19.9% and 7.4%, and 25.8% and 18.5%, for Health and Protection, respectively. Indeed, the proportion of reproductive health proposals among both health and protection proposals increased from 33.6% in 2009 to 53.4% in 2013, which translates to a 15.3% average increase in the number of relevant reproductive health proposals per year. Table 2 notes this information.

**Table 2:** Relevant reproductive health proposals, 2009-2013

	Annual Average	Overall change	Average change	Total	2009	2010	2011	2012	2013
Number of health proposals	672	-15.5%	-2.8%	3,358	814	712	600	544	688

Number of protection proposals	456	-34.4%	-9.3%	2,278	578	551	406	364	379

Number of relevant RH proposals	495	21.8%	10.0%	2,477	468	533	544	362	570

RH proposals (Health)	335	19.9%	7.4%	1,673	317	353	356	267	380

RH proposals (Protection)	161	25.8%	18.5%	804	151	180	188	95	190

Proportion of RH proposals among total sector proposals	44.6%	58.9%	15.3%	43.9%	33.6%	42.2%	54.1%	39.9%	53.4%

Proposals with full MISP implementation	28	92.9%	31.2%	139	14	29	26	43	27

Proposals with partial MISP implementation	249	16.5%	4.8%	1246	218	234	290	250	254

Proportion of full MISP proposals among total relevant RH proposals	6.0%	58.3%	39.5%	5.6%	3.0%	5.4%	4.8%	11.9%	4.7%

Proportion of partial MISP proposals among total relevant RH proposals	51.5%	-4.3%	2.4%	50.3%	46.6%	43.9%	53.3%	69.1%	44.6%

RH proposals addressing MNH	279	46.5%	13.2%	1,397	230	292	306	232	337

RH proposals addressing FP	74	37.0%	18.0%	370	46	86	74	101	63

RH proposals addressing HIV/STIs	186	-29.0%	-3.0%	930	214	240	211	113	152

RH proposals addressing RH-related GBV	227	34.2%	14.4%	1,136	196	251	265	161	263

RH proposals addressing general RH	130	-10.8%	0.0%	648	111	150	144	144	99

Proposals solely addressing non-RH GBV	81	-67.0%	-13.9%	404	97	138	65	72	32

Proposals addressing any type of GBV	308	0.7%	3.7%	1,540	293	389	330	233	295

Proportion of MNH proposals among total relevant RH proposals	56.7%	20.3%	5.1%	56.4%	49.1%	54.8%	56.3%	64.1%	59.1%

Proportion of FP proposals among total relevant RH proposals	15.7%	12.4%	23.3%	14.9%	9.8%	16.1%	13.6%	27.9%	11.1%

Proportion of STI/HIV proposals among total relevant RH proposals	37.5%	-41.7%	-12.4%	37.5%	45.7%	45.0%	38.8%	31.2%	26.7%

Proportion of RH-related GBV proposals among total relevant RH proposals	45.7%	10.2%	2.7%	45.9%	41.9%	47.1%	48.7%	44.5%	46.1%

Proportion of general RH proposals among total relevant RH proposals	27.1%	-26.8%	1.7%	26.2%	23.7%	28.1%	26.5%	39.8%	17.4%

Proportion of GBV-RH relevant proposals among total GBV proposals	74.0%	33.3%	9.0%	73.8%	66.9%	64.5%	80.3%	69.1%	89.2%

**Figure 5:**
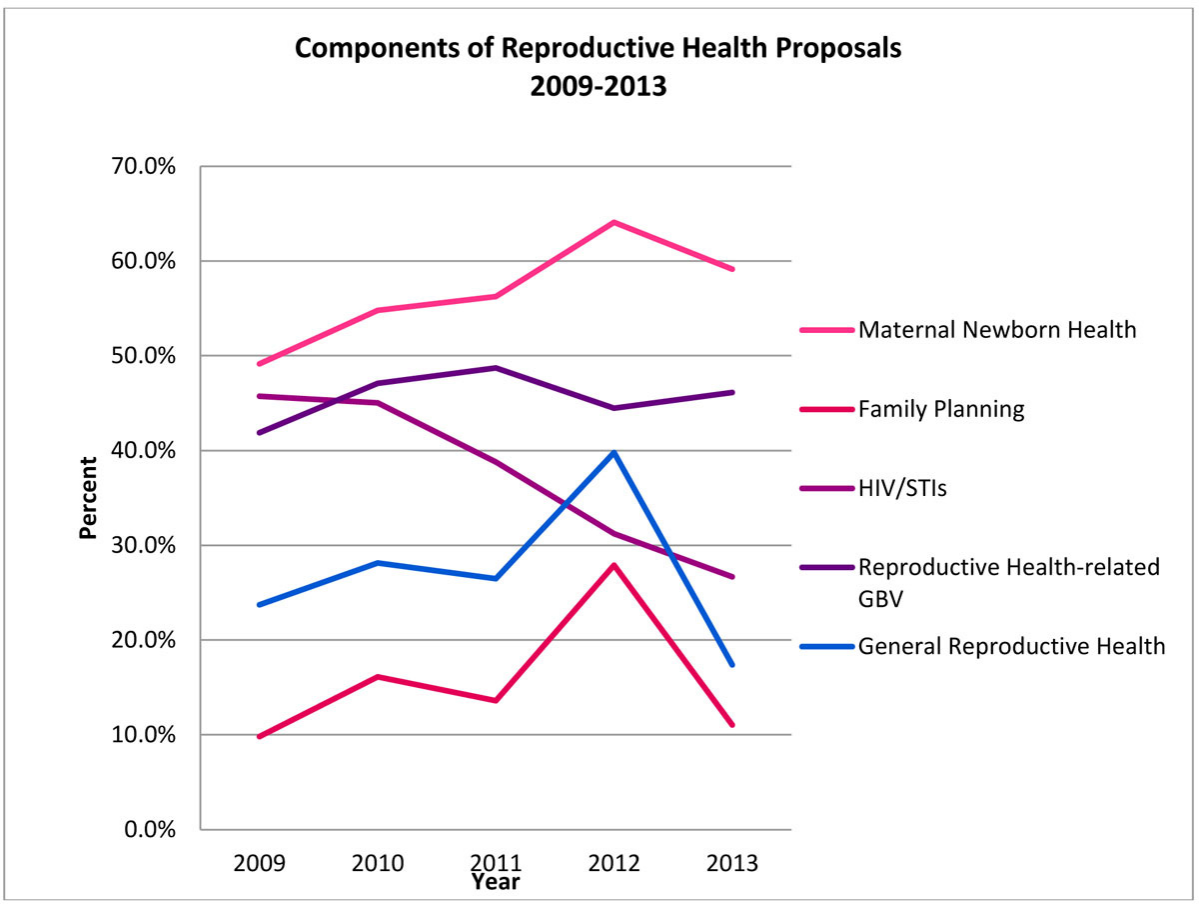


A closer examination of the types of reproductive health projects appealed during the five year period showed that activities within maternal newborn health comprised the largest proportion (56.4%), followed by GBV (45.9%), HIV/STIs (37.5%), general reproductive health (26.2%) and lastly, family planning (14.9%). As shown in Figure 5, the proportion of reproductive health proposals addressing maternal newborn health was 49.1% in 2009, 64.1% in 2012 and dropping to 59.1% in 2013. Family planning and general reproductive health activities showed a similar trend, at 9.8% and 23.7%, respectively in 2009. They both peaked at 27.9% and 39.8% in 2012, before decreasing to 11.1% and 17.4% in 2013, respectively. The proportion of proposals addressing HIV/STIs steadily decreased over the five year period, from 45.7% in 2009 to 26.7% in 2013. This marked an average annual decrease of 12.4% in proportions. The share of proposals that included reproductive health components as relevant to GBV grew marginally (average annual increase of 2.7%), with proportions ranging between 41.9% and 48.7% in 2009 and 2011 before decreasing to 44.5% and 46.1% in 2012 and 2013.

**Figure 6:**
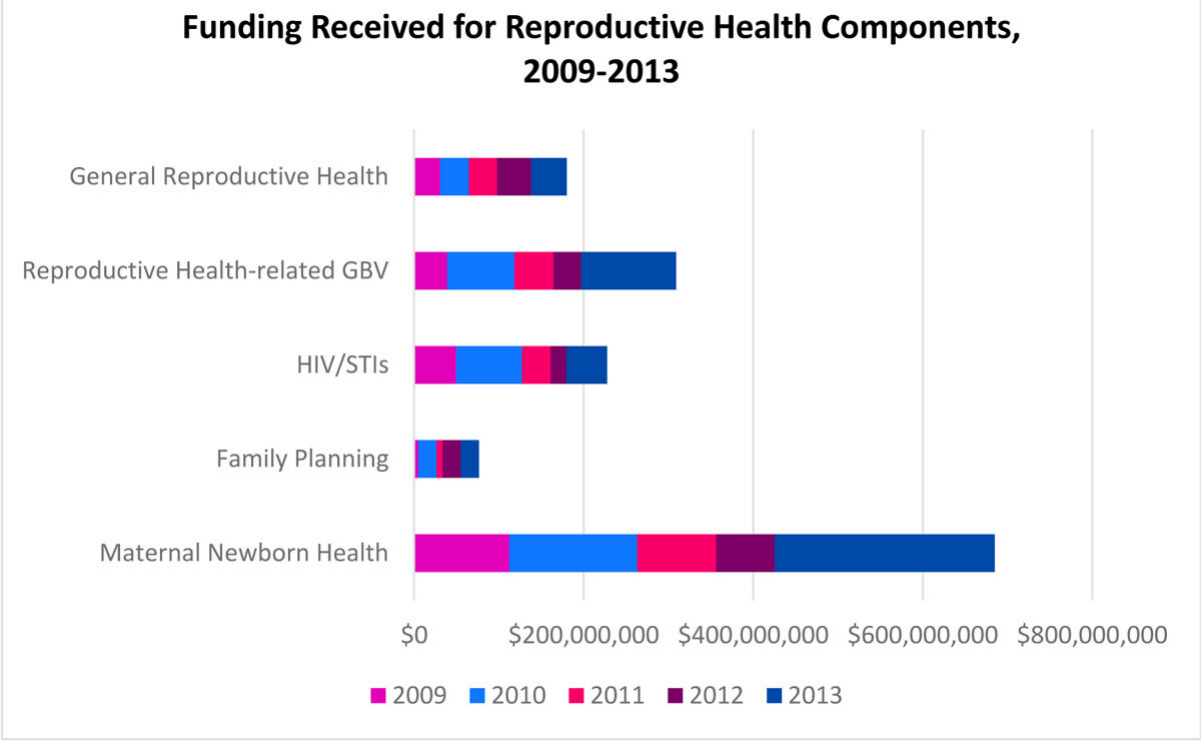


While a substantial number of proposals appealed to implement more than one component of reproductive health, when funding trends for the five reproductive health components were analyzed for the five year period, findings showed that maternal newborn health (55.7%) received the most funds as a proportion of requested funds, followed by general reproductive health (47.4%), family planning (47.4%), HIV/STIs (38.5%), and GBV (37.0%). In terms of absolute amounts, this translated to maternal newborn health receiving $684.8 million USD, general reproductive health $180.0 million USD, family planning a mere $76.3 million USD, HIV/STIs $227.6 million USD, and GBV $308.9 million USD. Hence, family planning received the least dollar amount among the reproductive health components. See Figure 6 for trends.

**Figure 7:**
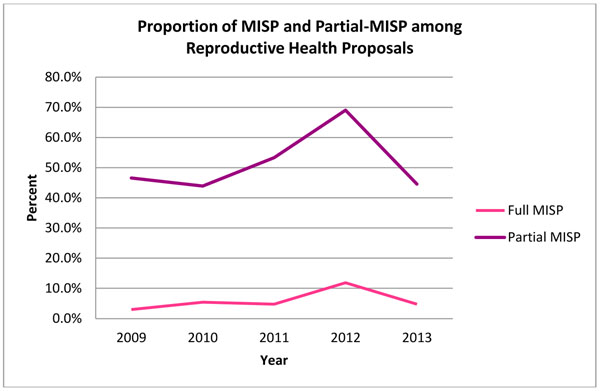


In terms of objectives and priority activities that comprise the MISP standard, the proportion of reproductive health proposals that noted complete MISP implementation (via explicit mention or a compilation of activities) increased from 3.0% in 2009 to 5.4% in 2010. This proportion jumped to 11.9% in 2012 before decreasing to 4.7% in 2013. Proposals that contained MISP activities in their partial form followed similar trends over the five year period, starting at 46.6% in 2009, jumping to 53.3% and 69.1% in 2011 and 2012, respectively, before decreasing to 44.6% in 2013. Figure 7 shows these trends. Overall, full MISP and partial MISP proposals comprised 5.6% and 50.3% of reproductive health proposals, respectively, with an average increase of 39.5% and 2.4% per year across the five years.

For GBV specifically, findings showed that 73.8% (1,136) of all GBV-related health and protection proposals (1,540) were relevant to reproductive health as defined by the IAFM. Non-reproductive health-related GBV proposals were those that solely appealed to implement non-health-related or broader GBV interventions (see Table 1). Proposals containing reproductive health-related GBV among all GBV proposals ranged from 64.5% (2010) to 89.2% (2013), with an average annual increase in share of 9.0%.

### Trends from 18 conflict-affected countries

In total, 3,988 health and 2,218 protection proposals were issued to aid the 18 conflict-affected countries between 2002 and 2013. This amounted to 54.7% of all issued sector proposals within the 12 year period. Among the 6,206 total proposals, 2,303 (37.1%) contained reproductive health activities. This marked an overall 400.0% increase from 2002 to 2013, with an average annual increase of 21.1%. In 2002, the proportion of health and protection proposals containing reproductive health activities was 22.5%, and in 2013, this was 59.5%.

**Figure 8:**
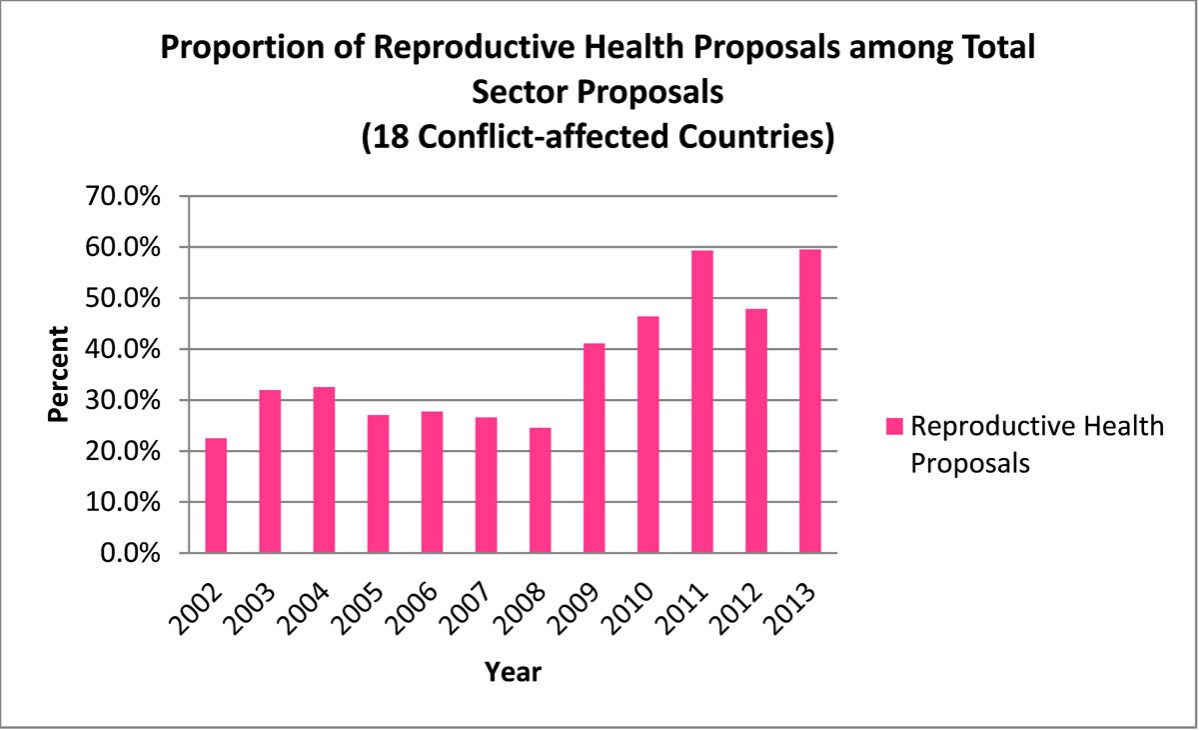


Within the 2009-2013 five year period where in-depth analysis was conducted, maternal newborn health activities comprised the majority of reproductive health proposals (59.4%), which increased from 47.3% of reproductive health proposals in 2002 to 65.1% in 2013. HIV/STI activities (40.2%) were second most mentioned, followed by reproductive health-related GBV (38.8%) and general reproductive health (23.0%), with family planning mentioned least (12.3%). Similar to the overall trends, all components were increasingly mentioned other than HIV/STIs, which dropped by an average of 9.2% per year. See Figure 8 for more information.

**Figure 9:**
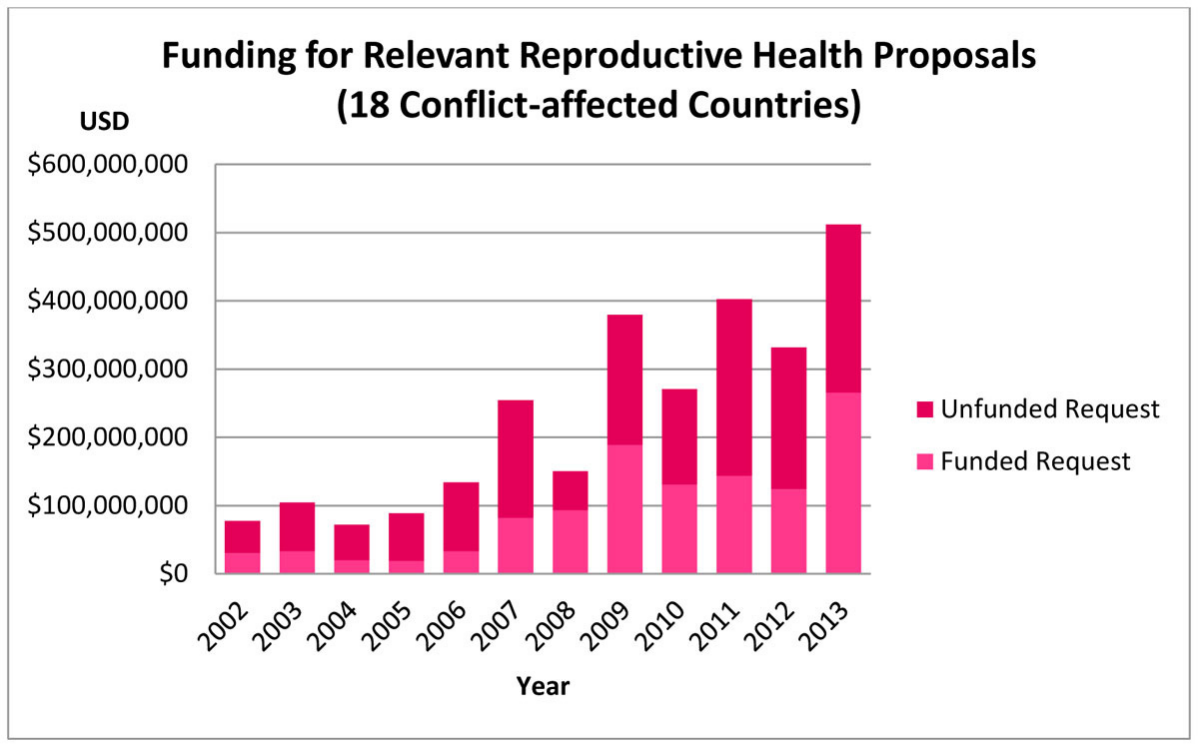


In terms of funding, the total request for reproductive health-related projects in the 18 conflict-affected countries was $2.777 billion USD for the 12 year period. The amount received was $1.165 billion; roughly 41.9% of the total request. The combined unfunded request was $1.612 billion USD. In 2002, the request to funding received was $77.3 million USD to $30.7 million USD (39.7%), while in 2013, this was $512.4 million USD to $265.8 million USD (51.9%). Similar overall proportions were observed across the 2002-2011 years of Patel et al.’s study: the total funding request was $1.933 billion USD, with $775.0 million USD received. This comprised 40.1% of the total request, leaving a total unfunded request of $1.158 billion USD [11]. Figure 9 contains additional information.

## Discussion

### Overall trends

Findings show increases in the number of proposals issued, proportion of proposals addressing reproductive health, and the amount of requested and received funds for reproductive health during the 12 year period examined: 2002-2013. An average annual increase of 21.9% in absolute numbers of proposals that include reproductive health and an average annual increase of 10.1% in the proportion of health and protection proposals that include reproductive health marks great strides in recognition of reproductive health needs in emergencies. When the type of appealing agencies are examined, they include the UN Population Fund and large humanitarian organizations, as well as local NGOs, Ministries of Health, and non-traditional reproductive health actors that are increasingly participating in appeals processes and are including reproductive health activities in their proposed programs. Such progress reflects the increasing participation of non-traditional actors in inter-agency funding mechanisms; awareness around the integration of reproductive health into global standards and guidance—such as the 2010 *Sphere Standards*, the 2009 Inter-agency Standing Committee *Health Cluster Guide*, and numerous other global policies and guidelines—as well as concerted advocacy that was undertaken by IAWG members over the last decade to ensure MISP implementation at the onset of an emergency in particular [3,18-22].

The total funding request over the 12 years amounted to $4.720 billion USD, of which $2.031 billion USD was received. While only 43.0% of the request was met, trends still show a 17.9% average annual increase in the proportion of requests received per year. Further, as a broader comparison over the 12 year period, total health sector proposals averaged 40.7% in funds received per total CAP requests and total protection sector proposals averaged 36.5%. Despite the data limited to the CAP, this shows that reproductive health in fact faired above Health and Protection averages, and was funded at only a slightly lower proportion than Water and Sanitation (averaging 44.1%). Overall, total sector proposals averaged 67.7% funding, with Health and Protection ranking fifth and tenth out of the eleven sectors (excluding “Sector Not Yet Specified”), respectively. Highest funded sectors were Food (averaging 85.7%), Coordination and Support Services (averaging 71.9%), and Multi-sector (averaging 68.9%) [23].

### In-depth analysis for 2009-2013

The proportion of reproductive health proposals appealing to implement maternal newborn health, family planning, and general reproductive health for 2009-2013 mirrors other IAWG global evaluation findings [22]. This shows increasing awareness towards the need to address reproductive health in humanitarian settings. However, when examining funds received, maternal newborn health received the most funding at $684.8 million USD, while family planning received the least, at $76.3 million USD. While family planning received comparable proportions of funding per request as compared to several other reproductive health components, it is important to emphasize the small number of proposals that in fact included family planning activities. Moreover, most proposals embedded family planning with other maternal newborn health or HIV prevention activities, possibly leading to an over-estimation of projects that substantially addressed family planning needs. Indeed, among the reproductive health components, proposals were least specific about the types of family planning services offered, and long-acting and permanent methods were seldom mentioned. All of these observations reflect the limited attention towards family planning in humanitarian settings, although possible caveats exist and are explained in further depth below.

Further, studies have shown that HIV has received substantial implementation and donor attention relative to other reproductive health components [4]. It is important to note that proposals with HIV/STI components tended to emphasize PMTCT as their primary focus versus comprehensive treatment and prevention activities. The five year analysis shows that proposals addressing HIV/STIs steadily decreased over the five year period, from 45.7% in 2009 to 26.7% in 2013. The proportion of received funds (38.5%) and absolute amounts ($227.6 million USD) were similarly less than those received for proposals that included maternal newborn health activities (55.7% and $684.8 billion USD). When examining reproductive health components via information available in Spreadsheet E for earlier years however, other observations are noteworthy. Health and protection proposals with HIV-related activities in fact comprised the largest share of reproductive health components from 2002-2008; only in 2009 did maternal newborn health proposals outrank HIV/STI proposals. In terms of funds received, HIV/STI proposals were most funded in 2003; however, the frequency with which antenatal and post-natal care was mentioned is the likely reason behind skewed funding to the maternal newborn health component. While prior year information was not included as part of the in-depth analysis given probable under-identification of proposed reproductive health activities where they were not noted in Spreadsheet E, the steady decline of HIV/STI proposals over 2009-2013 may thus be a result of changing need, integrated programming, or an equilibration of funding towards other reproductive health components. Alternative explanations may be a result of expanded programming by the Global Fund to Fight AIDS, Tuberculosis and Malaria (Global Fund) in fragile states, which may have contributed to HIV being considered a lower priority in humanitarian response [24,25]. Further, despite due emphasis on the continued need to ensure access to HIV prevention, care, and treatment, recognition that conflict and forced displacement does not necessarily lead to increased HIV prevalence may have also had its effects [26].

Indeed, the steady decline in the proportion of proposals containing HIV/STI activities (12.4% average decrease) was met with an increase in the absolute number of proposals containing reproductive health-related GBV activities (14.4% average increase). Increased attention to reproductive health-related GBV programming is also reflected in the larger requested and received amounts of funding for 2010-2013 where such funds exceeded those for HIV/STIs, family planning, and general reproductive health (aside from 2012) activities. The increase in proposals containing GBV could be the result of strong emphasis and collective advocacy to flag GBV as a salient and prevalent issue in crises, especially through UN Security Council Resolutions on Women, Peace and Security; the UN Secretary-General’s “UNiTE to End Violence against Women” campaign; and the work of the GBV Area of Responsibility, Gender Standby Capacity Project advisors, and other initiatives such as the gender marker [27-30].

In 2012, a major dip was observed in the proportion of sector proposals that included reproductive health. Absolute numbers of health and protection proposals decreased in 2012, as did the share of reproductive health, with a 33.5% reduction in the number of relevant sector proposals identified from the previous year. Among the relevant reproductive health proposals however, the share of maternal newborn health, family planning, and general reproductive health increased by 13.9%, 105.1%, and 50.3%, respectively. Further, 2012 showed the highest proportion of proposals noting full or partial MISP implementation (11.9% and 69.1%, respectively). Hence, the decrease in the absolute numbers of relevant proposals may possibly be explained by the fact that individual proposals addressed multiple reproductive health components or pledged to deliver a wider set of services. While the true reason is unknown, an important point to note is the value of examining the proportion of sector proposals with reproductive health components and the proportion of specific reproductive health components within relevant reproductive health proposals, rather than the mere absolute count that does not reflect the breadth and depth of programming.

Indeed, among relevant proposals, some activities were more often mentioned than others, even as proposals became increasingly detailed over the years. This presumably shows growing recognition and understanding of what a reproductive health response entails in emergencies, including appreciation of the MISP standard [22]. For MISP activities, among maternal newborn health services, emergency obstetric care (EmOC) and clean delivery kits were frequently mentioned. Despite reference to EmOC, abortion care of any kind was rarely mentioned; where it was mentioned in 13 proposals (two were withdrawn) was in the context of post-abortion care. Family planning as a component was least described although mentioned (as noted above); for HIV/STIs, PMTCT and safe transfusions were commonly mentioned in proposals where HIV activities were included. Clinical care for survivors of sexual violence and psychosocial care were most often mentioned for the reproductive health-related GBV component. Hygiene kits that encompassed menstrual hygiene were often listed for the general reproductive health component. The frequency that antenatal and post-natal care—neither a part of the MISP—were mentioned further contributed to the large share of maternal newborn health proposals overall, despite EmOC being more effective at reducing maternal morbidity and mortality [18]. In addition, nutrition was frequently mentioned in both health and protection proposals in 2013; specifically, the intersections between maternal newborn health and nutrition that skewed proposals and funded amounts for this component.

The lack of specification of family planning services and mention of abortion-related services may have been a result of agencies’ concerns over political and donor sensitivities, or legal restrictions in the case of the latter. Similarly, seemingly benign umbrella terms such as “maternal health” or even “emergency obstetric care” may have been used to denote more sensitive and specific services, or certain populations such as adolescents omitted, so that such services could be provided discreetly. The inability to accurately comprehend the services that are in fact provided at the field level is a major limitation of a key word search-based method. However, while projects for the Middle East North Africa region for instance, often did not mention reproductive health and GBV activities per se, a closer read of the project descriptions and names of the appealing agency could often shed light on the project scope, and where such information could be garnered, the proposals were marked under the relevant five reproductive health components. A further noteworthy observation is that while Marie Stopes International is a critical provider of abortion-related care and is increasingly responding in emergencies, the agency was only listed for seven projects over the 12 year period, reflecting a likely under-estimation of such service provision. While traditionally “development” actors are progressively responding in emergencies, there appears to remain a time lag in their participation in the FTS process, especially. Hence, the limited references to family planning and abortion-related services likely reflects a multitude of factors, although other IAWG global evaluation studies have indeed documented the disproportionate lack of availability of long-term and permanent methods of family planning and abortion services in particular [31].

In terms of cross-cutting populations, adolescents and persons with disabilities were mentioned across years, but in very few proposals. These proposals were typically entirely dedicated to the specific population, showing limited mainstreaming.

### Trends from 18 conflict-affected countries

Patel’s study found that the average annual ODA disbursed for reproductive health to 18 conflict-affected countries from 2002 to 2011 was $747.0 million USD. While this study calculated the total amount at $775 million USD for the same time period, the discrepancies are explainable from the different units of analyses, as well as several classification differences in what constituted reproductive health activities. Key differences between the 18 countries and the overall trend are the proportions of specific reproductive health components within relevant proposals for 2009-2013: HIV/STIs proposals held a higher share among proposals appealed for the 18 countries, ranking second overall. Family planning however, mirrored overall trends and was mentioned in a mere 12.3% of relevant reproductive health proposals.

## Limitations

Several limitations exist in this study. First, the analysis is solely based on desk research of proposals submitted through the FTS. Hence, the analysis is only as accurate in-so-far as agencies voluntarily report their planned activities. Some of the Gulf States and Islamic charities are yet to actively participate in the FTS, which misses their relevant efforts [32]. Some reproductive health-related activities may have also been missed due to human error; if activities were not mentioned in the proposal or were subsumed under vague descriptions due to political sensitivities; or if modifications were made beyond what was captured in appeals revision processes. This study looked at projected programming and funding for reproductive health as addressed in health and protection proposals submitted to the FTS. Thus, findings indicate practitioner and donor recognition of the need for reproductive health in emergencies and cannot speak to actual implementation or the quality of services provided. Limitations of the desk research further apply to the amount of received funds; if contributions were not reported through the FTS, the information was not captured in this study.

Second, duplicate appeals are included in the context of revised appeals to account for evolving programming with time. Duplicate programs have also been included where an umbrella organization—typically a UN agency—has appealed for the same project that is in fact implemented by international and national partners. Their inclusion, however, brings about discrepancies in funds received. While appeals include multiple agency requests for the same project—especially where partnership arrangements have been made—when funds are received, they are most likely reflected as received by the umbrella agency, and not by all of its sub-grantees [33]. The unfunded request will thus be systematically overestimated across years.

Third, and related to duplicate counts, since it was not possible to untangle the amount of money that was distributed across different reproductive health components where proposals appealed to implement multiple components, the study assumed that agencies proportionally allocated requested and received funds across the number of mentioned reproductive health components for the 2009-2013 analysis. This may contribute some inaccuracies if proposals focused on certain components over others that they addressed; however, the authors deemed that this would be more accurate than not weighting the proposals at all.

Fourth, while originally, all proposals across 2002-2008 were planned for an in-depth review, full narrative appeals were only available from 2005, and all project descriptions (proposals) from 2009. Hence, to conduct a consecutive 5-year analysis, the study team added data from 2013 for analysis of 2009-2013.

Fifth, to prevent inflated and duplicative counts within reproductive health thematic areas, activities were categorized into five thematic areas per the MISP and comprehensive reproductive health (minus the MISP) for the 2009-2013 in-depth analysis. The counts for each thematic area are therefore likely to be under-represented.

Sixth, as only health and protection proposals were analyzed, any relevant activities appealed in other sectors/clusters would have been missed. These include large-scale infrastructural improvements that could support EmOC transfers, menstrual hygiene as included under non-food item distributions, related projects under Water and Sanitation, or multi-sectoral projects that were not mentioned in Health/Protection. However, protection proposals were analyzed to minimize under-representation of GBV-related projects.

Seventh, given challenges to linking donor contributions to exact appeals through the FTS, only aggregate funding was examined. This limits comparisons with the ODA analysis where the type of donor was examined in-depth.

## Conclusions

This study is the first in-depth analysis that reviewed commitments to reproductive health through project and funding data reported to the FTS. Findings show that more agencies are appealing to implement reproductive health programs in terms of the number of issued proposals and funding requested over the 12 year period. The absolute amounts received by agencies to implement relevant reproductive health activities also increased during this time. While such developments are welcome, based on project descriptions and the scope of this analysis, proposals show comparatively limited attention and programming for family planning services and to abortion care in particular. At the intersections of the International Conference on Population and Development plus 15 and the Post-2015 Agenda, the timing is opportune to scale-up attention and access to such critical, life-saving services in humanitarian response.

## List of abbreviations used

ANC: Antenatal care; ARV: Antiretroviral ; CAP: Consolidated Appeals Process; CHAP: Common Humanitarian Action Plan; CRS: Creditor Reporting System; EmOC: Emergency obstetric care; FTS: Financial Tracking Service; GBV: Gender-based violence; IAFM: *Inter-agency Field Manual on Reproductive Health in Humanitarian Settings;* IAWG: Inter-agency Working Group on Reproductive Health in Crises; MISP: Minimum Initial Service Package; OCHA: Office for the Coordination of Humanitarian Affairs; ODA: Official Development Assistance; PMTCT: Prevention of mother-to-child transmission; RH: Reproductive Health; STI: Sexually transmitted infection; UN: United Nations; USD: United States dollar; WRC: Women’s Refugee Commission.

## Competing interests

The authors declare no competing interests.

## Authors' contributions

MT developed the study methodology, with contributions from PP. KS primarily conducted the data analysis, with help from SR. MT conceptualized the paper and was principal author; KS, PP, and SKK contributed to the writing process. All authors reviewed and approved the final text.

## Endnotes

^a^ Columns from FTS’ spreadsheets that were retained included: year; country/region; country/region code; appeal type; appeal title; project code; title; appealing agency; original requirements USD; revised requirements USD; funding USD; % covered; unmet requirements USD; and uncommitted pledges USD.

^b^ Overall, the analysis team spent between 30 seconds and 10 minutes to assess and categorize each proposal, depending on their relevance to reproductive health. The principal investigator conducted periodic, random comparison checks to ensure consistency in data analysis across the two analysts and over time.

^c^ The objectives of the MISP are to: 1) ensure effective coordination; 2) prevent sexual violence and manage its consequences; 3) reduce HIV transmission; 4) prevent excess maternal and neonatal morbidity and mortality; and 5) plan for comprehensive reproductive health services.

## Declarations

This article has been published as part of *Conflict and Health* Volume 9 Supplement 1, 2015: Taking Stock of Reproductive Health in Humanitarian Settings: 2012-2014 Inter-agency Working Group on Reproductive Health in Crises' Global Review. The full contents of the supplement are available online at http://www.conflictandhealth.com/supplements/9/S1. Funding for this supplement was provided by the MacArthur Foundation.
